# A review of the effect of skin pigmentation on pulse oximeter accuracy

**DOI:** 10.1088/1361-6579/acd51a

**Published:** 2023-06-01

**Authors:** Raghda Al-Halawani, Peter H Charlton, Meha Qassem, Panayiotis A Kyriacou

**Affiliations:** 1 Research Centre for Biomedical Engineering, City, University of London, London, United Kingdom; 2 Department of Public Health and Primary Care, University of Cambridge, Cambridge, United Kingdom

**Keywords:** pulse oximetry, photoplethysmography, COVID-19, skin pigmentation, accuracy

## Abstract

*Objective*. Pulse oximetry is a non-invasive optical technique used to measure arterial oxygen saturation (SpO_2_) in a variety of clinical settings and scenarios. Despite being one the most significant technological advances in health monitoring over the last few decades, there have been reports on its various limitations. Recently due to the Covid-19 pandemic, questions about pulse oximeter technology and its accuracy when used in people with different skin pigmentation have resurfaced, and are to be addressed. *Approach*. This review presents an introduction to the technique of pulse oximetry including its basic principle of operation, technology, and limitations, with a more in depth focus on skin pigmentation. Relevant literature relating to the performance and accuracy of pulse oximeters in populations with different skin pigmentation are evaluated.* Main Results*. The majority of the evidence suggests that the accuracy of pulse oximetry differs in subjects of different skin pigmentations to a level that requires particular attention, with decreased accuracy in patients with dark skin. *Significance*. Some recommendations, both from the literature and contributions from the authors, suggest how future work could address these inaccuracies to potentially improve clinical outcomes. These include the objective quantification of skin pigmentation to replace currently used qualitative methods, and computational modelling for predicting calibration algorithms based on skin colour.

## 1. Introduction

**Figure 1.**
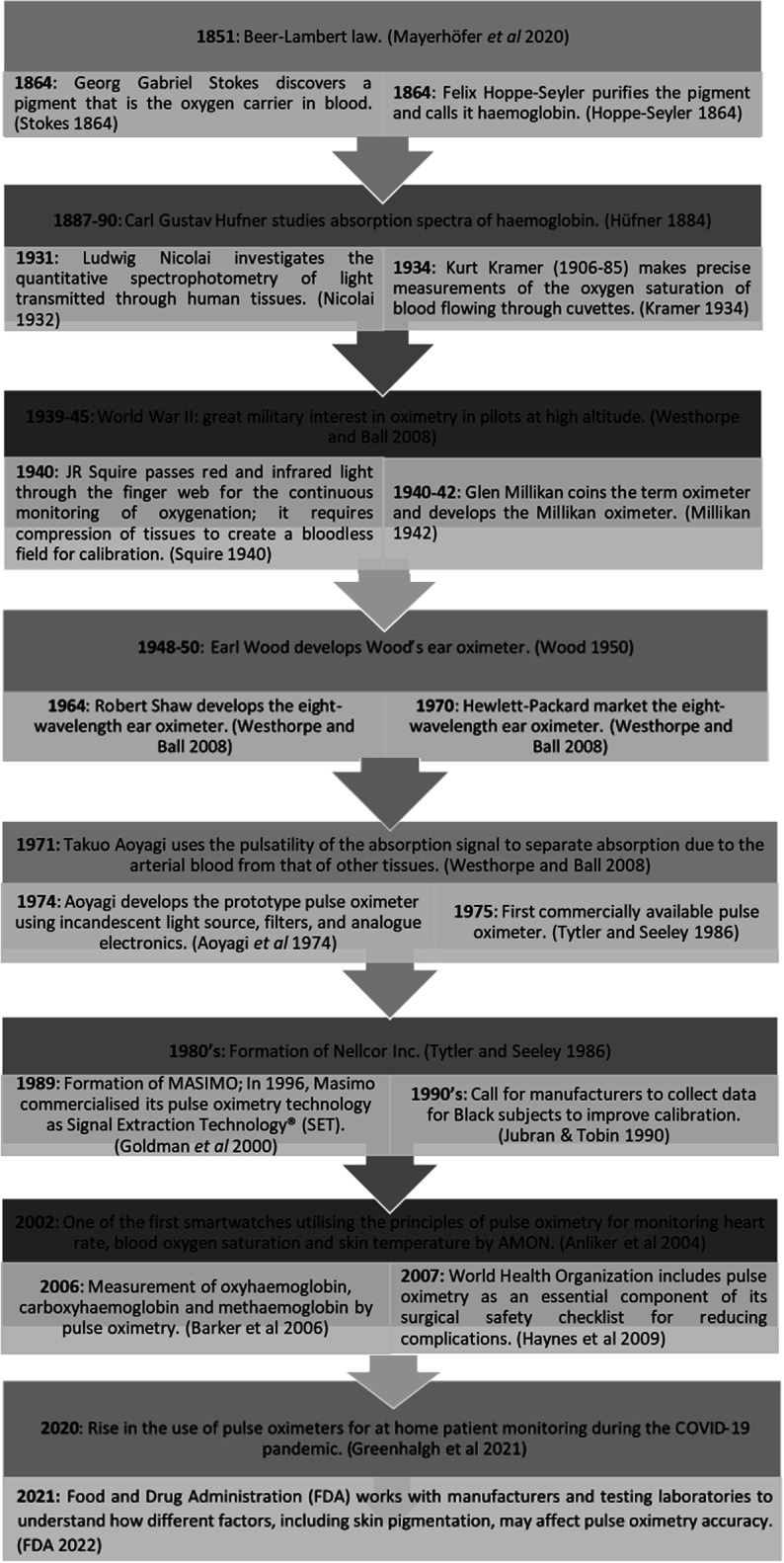
Key events in the history of pulse oximeter technology.

A pulse oximeter is a non-invasive and low cost, optical device, based on a two wavelength photoplethysmography (PPG) system, used for the continuous monitoring of arterial oxygenation. Pulse oximetry has been found to work on the finger, the ear, the bridge of the nose, the nasal septum, the temple over the temporal artery, and on the foot or palm in infants. Arterial oxygen saturation measurement plays a central role in the diagnosis of sleep-related respiratory disorders such as obstructive sleep apnoea (OSA), monitoring and treatment of respiratory diseases by detecting hypoxaemia, assisting the titration of supplemental oxygen treatment in preterm neonates, and many more (Kyriacou and Allen 2021). Pulse oximetry is now routinely used in a wide range of clinical settings, ranging from hospital care to primary care to home monitoring (Kelleher 1989, Morris *et al* 1989, Tremper and Barker 1989, Wilkins *et al* 1989, Falconer and Robinson 1990, Eichhorn 1993, Lawless 1994, Wahr *et al* 1995, Dumas *et al* 1996, Shah *et al* 2012). Pulse oximeters have been shown to be an effective tool for identifying the need for hospitalisation in initially non-severe and possibly high-risk COVID-19 patients, or for discharging known or suspected Covid patients (Levitan 2020, Luks and Swenson 2020, Philip *et al* 2020, Quaresima and Ferrari 2020, Rodriguez 2020, Teo 2020, Tobin *et al* 2020, Shah 2020, Brouqui 2021, Cajanding 2021, Cysewska-Sobusiak 2021, Gootenberg *et al* 2021, Greenhalgh *et al* 2021, Lancet *et al* 2021, Michard *et al* 2021, Ngiam *et al* 2021, Wilson-Baig *et al* 2021). Hence, pulse oximetry is now widely used in clinical and consumer settings (Teo 2020), underlining the need to ensure it is as accurate and reliable as possible. Figure 1 provides a succinct summary of the key events in the history of pulse oximeter technology.

There are rising concerns of the differing performance of pulse oximetry in patients with different skin pigmentations (Wang and Poh 1985, Cecil *et al* 1988, Gabrielczyk and Buist 1988, Ries *et al* 1989, Cahan *et al* 1990, Jubran and Tobin 1990, Ralston *et al* 1991, Volgyesi and Spahr-Schopfer 1991, 1992, Lee *et al* 1993, Bothma *et al* 1996, Adler *et al* 1998, Bickler *et al* 2005, Reuss 2005, Feiner *et al* 2007, Witting and Scharf 2008, Foglia *et al* 2017, Ebmeier *et al* 2018, Murphy and Omar 2018, Guber *et al* 2019, Smith and Hofmeyr 2019, Badgujar *et al* 2020, Dyer 2020, Michael *et al* 2020, Philip *et al* 2020, Tobin *et al* 2020, Baker *et al* 2021, Hunasikatti 2021, Philip *et al* 2021, Sjoding *et al* 2021, Todd 2021, Valbuena *et al* 2021, Vesoulis *et al* 2021, Whitehead 2021, Wiles *et al* 2021, Wong *et al* 2021, Baker and Wilson 2022, Bangash *et al* 2022, Burnett *et al* 2022, Crooks *et al* 2022, Fawzy *et al* 2022, Ferrari *et al* 2022, Henry *et al* 2022, Holder and Wong 2022, Knight *et al* 2022, Kyriacou *et al* 2022, Okunlola *et al* 2022, Shi *et al* 2022, Tobin and Jubran 2022, Wiles *et al* 2022). The concept that the accuracy of pulse oximetry may differ between people of different skin pigmentations is not new. Rather, its importance has increased in the Covid-19 pandemic (Greenhalgh *et al* 2021), since the deterioration of blood oxygenation is one of the primary symptoms of severe Covid-19. The limitations of pulse oximetry, including the impact of ambient light, difficulties in obtaining measurements during low perfusion and motion, etc, are known (Jubran 2015) and guidance on how to minimise their effect is available (GOV.UK 2021). However, the increased mortality rate amongst ethnic minority patients since 2020 from the Covid-19 outbreak has raised the question of whether differential accuracy of pulse oximetry due to skin pigmentation may be a contributing factor to this health inequality. Consequently, this review aims to summarise the literature on the effect of skin pigmentation on pulse oximeter accuracy, and based on this, recommend solutions for minimising these effects.

## 2. Principles of pulse oximetry

### 2.1. The gold standard: blood gas analysis

Blood gas analysis is currently the gold standard for blood oxygenation measurement. It was the only method available before the introduction of non-invasive blood oxygenation measurements. Blood gas analysers consist of three electrodes that measure pH, partial pressure of carbon dioxide (PaCO_2_) and partial pressure of oxygen (PaO_2_) at 37 °C using extracted blood samples (Severinghaus 2006).

Two main indicators are used to reveal the amount of oxygen present in blood, namely functional arterial oxygen saturation (SaO_2_) and fractional arterial oxygen saturation (FrO_2_). Both indices represent the percentage content of oxygen-bound haemoglobin (HbO_2_) against the total haemoglobin present. This is typically performed by measuring the concentration of the different haemoglobin species of interest. SaO_2_, expressed as a percentage, is then determined by the ratio of HbO_2_ concentration over the sum concentration of HbO_2_ and deoxyhaemoglobin (HHb) in blood

\begin{eqnarray*}{{\mathrm{SaO}}}_{2}=\displaystyle \frac{[{{\mathrm{HbO}}}_{2}]}{\left[{{\mathrm{HbO}}}_{2}\right]+[{\mathrm{HHb}}]}.\end{eqnarray*}

Similarly, the fractional saturation, FrO_2_, is determined by the proportion of oxygenated haemoglobin over the total concentration of all haemoglobin species

\begin{eqnarray*}{{\mathrm{FrO}}}_{2}=\displaystyle \frac{[{{\mathrm{HbO}}}_{2}]}{\left[{{\mathrm{HbO}}}_{2}\right]+\left[{\mathrm{HHb}}\right]+\left[{\mathrm{COHb}}\right]+[{\mathrm{MetHb}}]},\end{eqnarray*}

where [COHb], and [MetHb] are respectively the concentrations of carboxyhaemoglobin and methaemoglobin. FrO_2_ is a more accurate method for determining oxygenation of blood, especially when the presence of other dyshaemoglobins (haemoglobins that do not bind to oxygen) is suspected due to particular physiological conditions.

### 2.2. Peripheral oxygen saturation

Pulse oximetry is non-invasive, low-cost, safe and is currently performed routinely on all surgical patients during admission, intraoperatively and postoperatively. It is more readily available than arterial blood gas analysis for the measurement of oxygen saturation and provides continuous monitoring. It has largely replaced the blood gas method in many clinical settings, unless carbon dioxide or acid-base status is specifically required (Smith and Hofmeyr 2019).

**Figure 2.**
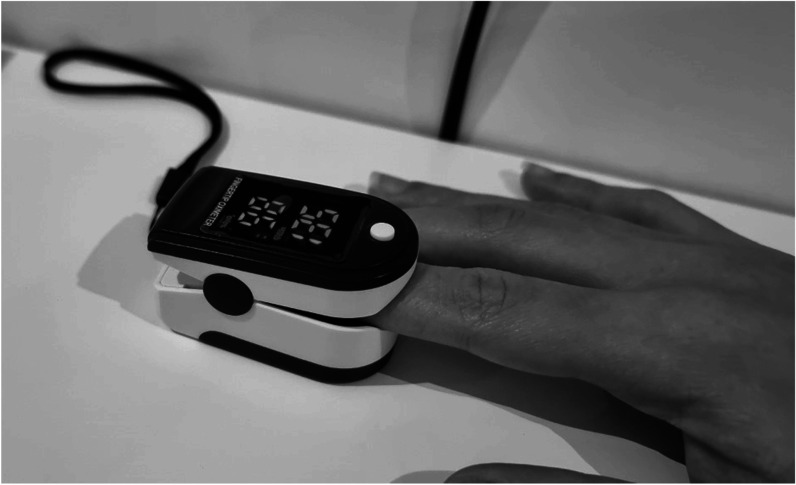
A pulse oximeter device for the non-invasive measurement of arterial oxygen saturation, available from pharmacies for home monitoring.

**Figure 3.**
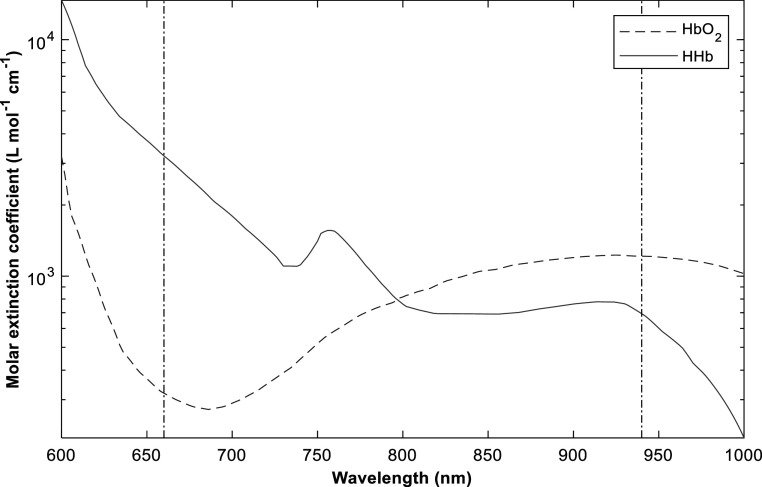
Absorption spectra of oxygenated (HbO_2_) and deoxygenated (HHb) haemoglobin between the visible and near-infra-red region. Molar extinction coefficients of both haemoglobin species are shown with respect to the wavelengths of interest in pulse (Kyriacou and Allen 2021). Reprinted from Kyriacou *et al* 2022, Copyright (2022), with permission from Elsevier.

Pulse oximeters (figure 2) estimate functional arterial oxygen saturation by measuring changes in light absorbance in the arterioles over time, as they contain a higher concentration of HbO_2_ relative to HHb. The photoplethysmogram (PPG) is used to detect blood volume changes and to differentiate between absorbance of arterial blood and other absorbers (skin, bone, venous blood). A good choice of wavelength is where there are large differences in the extinction coefficients (*ε*) of HbO_2_ and HHb (Webster ) (figure 3). Another criterion for the wavelength selection is the relative flatness of the absorption spectra around the chosen wavelength (Moyle 1994, Mannheimer *et al* 1997b). The two conventional wavelengths used in pulse oximetry are the 660 nm (red) and 940 nm (near infra-red). Measurement of arterial oxygen saturation by pulse oximetry is denoted by SpO_2_, while the term SaO_2_ is generally reserved for arterial oxygen saturation measured by blood gas analysis in extracted blood (Nitzan *et al* 2020).

The Beer–Lambert law forms the basis of light absorption measurements where the effects of scattering are negligible (Mannheimer *et al* 1997a). It relates the attenuation of light to the properties of the material through which the light is travelling, which is assumed to be an absorbing-only medium (Nitzan *et al* 2020):

\begin{eqnarray*}{I}_{t}={I}_{0}{{\mathrm{e}}}^{\left(-\varepsilon Cd\right)},\end{eqnarray*}

where ${I}_{t}$ is the intensity of transmitted (i.e. received) light, ${I}_{0}$ is the intensity of the incident light, *d* is the optical pathlength of the tissue under investigation (the source-detector distance in reflectance mode), *ε* is the molar extinction coefficient of tissue layers and chromophores such as haemoglobin, melanin, water, etc, and *C* refers to their

#### 2.2.1. Concentrations

The Beer–Lambert law does not account for the scattering of light as it passes through tissue. To address this, the Beer–Lambert law can be modified to account for scattering by: (i) introducing a term, *G*, representing ‘light-loss due to scattering’, and (ii) replacing the pathlength, *d*, with the optical pathlength, $l,$ where the optical pathlength represents the actual distance travelled by light, which is longer than the pathlength due to scattering (Nitzan *et al* 2020). The modified Beer–Lambert law is (Nitzan *et al* 2020):

\begin{eqnarray*}{I}_{t}={I}_{0}{{\mathrm{e}}}^{(-G-\varepsilon Cl)}.\end{eqnarray*}

**Figure 4.**
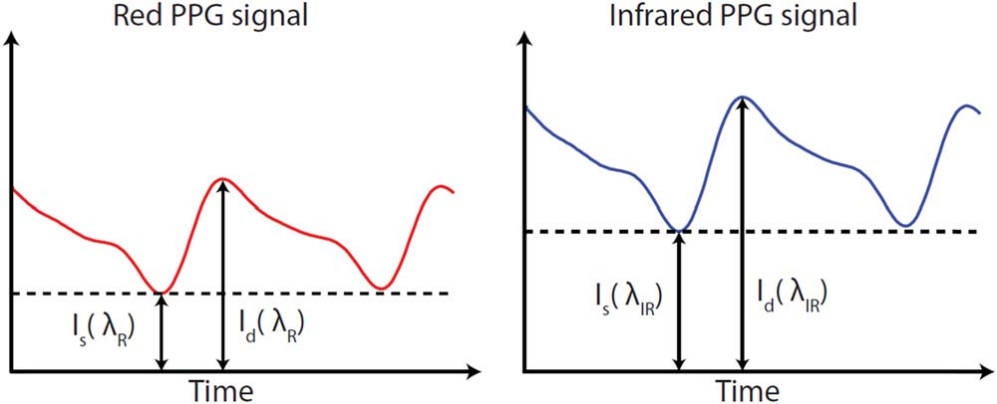
Intensity of red and infra-red PPG signals during diastolic (${I}_{d}$) and systolic (${I}_{s}$) absorption.

The modified Beer–Lambert law can be used to estimate SpO_2_ as described in (Nitzan *et al* 2020). To do so, measurements of the intensity of transmitted light, *I*_*t*_, are extracted at points of minimum and maximum absorptions during the cardiac cycle (see figure 4). This produces a pair of equations which are combined to eliminate ${I}_{0}.$ It is assumed that *G* is approximately constant throughout a cardiac cycle, thereby eliminating *G* from the resulting equation. *I* is also assumed to be constant throughout a cardiac cycle. This produces the following equation relating the PPG measurements (*I*_*D*_ *and I*_*S*_ at maximum and minimum absorptions respectively) to the maximal change in haemoglobin concentration during a cardiac cycle, Δ*C* (Nitzan *et al* 2020):

\begin{eqnarray*}\left({I}_{D}-{I}_{S}\right)/{I}_{S}=\varepsilon {\mathrm{\Delta }}Cl.\end{eqnarray*}

This equation is applied twice, once to *I*_*D*_ and *I*_*S*_ extracted from a PPG signal obtained using a red light, and then again to *I*_*D*_ and *I*_*S*_ extracted from a PPG signal obtained using infra-red light. Assuming that Δ*C* is approximately equal at the two wavelengths, the two resulting equations are combined to produce the ‘ratio of ratios’, *R* (Nitzan *et al* 2020):

\begin{eqnarray*}R\equiv \displaystyle \frac{{\left[({{I}}_{D}-{{I}}_{S})/{{I}}_{S}\right]}_{660}}{{\left[({{I}}_{D}-{{I}}_{S})/{{I}}_{S}\right]}_{940}}=\displaystyle \frac{{\varepsilon }_{660}\,{{l}}_{660}}{{\varepsilon }_{940}\,{{l}}_{940}}\end{eqnarray*}

where the subscripts ‘660’ and ‘940’ indicate the two wavelengths. The extinction coefficients can then be expressed in terms of blood oxygen saturation, SaO_2_, using (Nitzan *et al* 2020):

\begin{eqnarray*}\varepsilon ={\varepsilon }_{O}{\mathrm{SaO}}_{2}+{\varepsilon }_{D}\left(1-{\mathrm{SaO}}_{2}\right),\end{eqnarray*}

where *ε* *_O_* and *ε* *_D_* are extinction coefficients for oxygenated and deoxygenated blood respectively. Substituting equation (7) into equation (6), and rearranging, an expression is obtained relating SpO_2_ to known quantities (*ε*) and the measured *R*:

\begin{eqnarray*}{\mathrm{SpO}}_{2}=\frac{{\varepsilon }_{{D}_{660}}-R\left(\displaystyle \frac{{l}_{940}}{{l}_{660}}\right){\varepsilon }_{{D}_{940}}}{R\left(\displaystyle \frac{{l}_{940}}{{l}_{660}}\right)\left({\varepsilon }_{{O}_{940}}-{\varepsilon }_{{D}_{940}}\right)+\left({\varepsilon }_{{D}_{660}}-{\varepsilon }_{{O}_{660}}\right)}.\end{eqnarray*}

**Figure 5.**
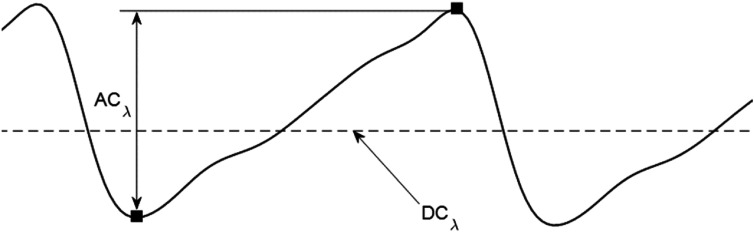
PPG parameters used for the calculation of the ratio of ratios (*R*). The AC amplitude at a generic wavelength $\lambda $ is obtained from the difference between minimum and maximum absorption (squares) during the cardiac cycle. The DC component is the average light intensity. These two parameters are extracted from red and infra-red wavelengths for calculation of the ratio of ratios (*R*) in equation (5) (Kyriacou and Allen 2021). Reprinted from Kyriacou *et al* 2022, Copyright (2022), with permission from Elsevier.

Note that all these values are known constants expect *R* and the optical pathlengths at the two wavelengths. Consequently, most modern pulse oximeters convert measurements of *R* to SpO_2_ using a ‘look-up table’ (Webster 1997b), based on the assumption that *l*_940_ */l*_660_ remains constant between individuals, using the following equation:

\begin{eqnarray*}{R}=\displaystyle \frac{\tfrac{{\mathrm{A}}{{\mathrm{C}}}_{660}}{{\mathrm{D}}{{\mathrm{C}}}_{660}}}{\tfrac{{\mathrm{A}}{{\mathrm{C}}}_{940}}{{\mathrm{D}}{{\mathrm{C}}}_{940}}},\end{eqnarray*}

where [${{\mathrm{ac}}}_{660}$] and [${{\mathrm{dc}}}_{660}$] represent the alternating current and direct current from tissue layers at 660 nm (a red wavelength) respectively, and likewise for [${{\mathrm{ac}}}_{940}$] and [${{\mathrm{dc}}}_{940}$] at 940 nm (an infra-red wavelength). Manufacturers calibrate pulse oximeters empirically by correlating the measured ratio (*R*) of AC/DC signals (figure 5). Most modern pulse oximeters convert these ratios to SpO_2_ using a ‘look-up table’ (Webster 1997b).

This approach assumes that the relationship between *R* and SaO_2_ is constant across different individuals, which would be a reasonable assumption if the inter and intra variability of skin components in individuals are disregarded. For instance, the magnitude of absorbance and the optical pathlength is influenced by the presence of melanin in tissue. Since there is a directly proportional relationship between melanin concentration and *μ* *_a_*, the value of *μ*_*a*_epidermis_ in individuals with dark skin is greater than in individuals with fair skin, which results in increased light absorption (Bashkatov *et al* 2000). Additionally, the type of light scattering, i.e. Rayleigh and Mie scattering, is determined by the size of the scatterer relative to the selected wavelength, which has been found to be larger for individuals with darker skin (Zonios *et al* 2001). As a result, photons would have a tendency to travel in a more forward direction (Mie scattering) in individuals with dark skin. Hence, current pulse oximeter calibrations may not be captured for wide populations, which may be contributing to the differing performance of pulse oximeters in patients with different skin pigmentations.

### 2.3. Modalities of pulse oximetry

There are two geometrical configurations of pulse oximetry, namely transmittance and reflectance mode. In transmittance mode PPG, the source and detector are placed opposite to one another across the surface of the region of interest (ROI), most commonly the finger. Therefore, the optical path length is affected by the amount of light scattering within the tissue, as well as the tissue thickness, which alters the distance between the fixed source and detector. However, in reflectance mode PPG, the source and detector are placed adjacent to each other and the light detected is back scattered and reflected within the tissue. Reflectance PPG offers greater flexibility than transmittance PPG in regards to the position of the sensors in different anatomical locations, such as the forehead. The source-detector separation can be altered by changing the distance between the source and detector, resulting in differences in the behaviour of light. For instance, if the desired outcome is to achieve maximum photon penetration depth, the source-detector separation can be increased in order to see the interaction of light with deeper tissue. In both modalities, the light-tissue interactions that take place vary depending on the wavelength of the light source, which alters the optical properties of the tissue components.

### 2.4. Limitations of pulse oximetry

It is important to acknowledge the limitations and assumptions of pulse oximetry before delving further into the impact and/or possible causes of skin pigmentation on the accuracy of pulse oximetry measurement. This is because inaccurate measurements may result from a combination of certain conditions, than in the presence of either condition alone.

As previously stated, pulse oximeters measure functional oxygen saturation, which as a result, assumes that the only haemoglobin species bounding with oxygen and present in blood are deoxygenated and oxygenated haemoglobin. However, other dyshaemoglobins such as carboxyhaemoglobin (COHb) and methaemoglobin (MetHb), may be present in the blood due to higher concentrations or partial pressures of carbon monoxide and/or other gases and chemical compounds). The most common case is carboxyhaemoglobin, which is a molecule of haemoglobin that has bound reversibly with carbon monoxide (CO). In the visible range of the light spectrum, COHb presents a very similar absorption profile to oxyhaemoglobin, thus making it very difficult to distinguish between the two haemoglobins. This does not cause any particular concern in healthy subjects, but, in particular cases such as carbon monoxide poisoning or smokers, COHb may be present in higher concentrations in blood. This could potentially result in erroneous SpO_2_ estimations, although they have been seen to minimally effect measurements of SpO_2_ (Feiner *et al* 2013).

Furthermore, accurate estimation of blood oxygen saturation may be compromised under conditions of poor blood perfusion. To estimate SpO_2_ accurately, pulse oximeters rely on the optical measurements of arterial pulsations (i.e. PPG signals) within the tissue bed. However, these pulsations only make up 2%−5% of the total optical absorption profile recorded and are significantly dependent on an adequate blood flow/supply to the measurement area. When blood flow to certain anatomical areas is compromised due to, for instance, hypovolemia, hypotension or hypothermia, the arterial pulsations recorded by the pulse oximeter may be significantly reduced or disappear, causing inaccurate SpO_2_ measurements. A reasonable solution is to move the sensor from a peripheral site, such as the finger to a more central location with adequate perfusion. However, this may be not ideal in cases of severe critical illness such as patients experiencing a high degree of hypoperfusion. Another cause of falsely low SpO_2_ is changes in venous volume due to venous pulsations, which can result from a number of scenarios including tightly placed finger probes, or again, in critically ill-patients suffering from heart complications.

Pulse oximeters are well known to provide inaccurate readings when the light absorption profile of red and/or infra-red light is corrupted. Nail polish and artificial fingernails have been reported to affect pulse oximeter readings measured at the fingertips. More specifically, dark colours of nail polish such as black or blue can cause false readings and lead to inaccuracies (Coté *et al* 1988, Çiçek *et al* 2011, Yönt *et al* 2014). As this is a well-known limitation of pulse oximetry, removing nail polish or changing the measurement site can eliminate this particular issue. Contrarily to the popular understanding that nail polish causes false SpO_2_ readings, some studies have determined a limited or insignificant impact on SpO_2_ readings (Rodden *et al* 2007, Yamamoto *et al* 2008, Balaraman *et al* 2020). However, the reported differences in the literature may be due to methodological differences and more standardised studies are required to assess the existence of such interference across different pulse oximeter manufactures, nail polish colours, and larger populations.

Another limitation is the effect of light pollution (ambient light) (Mathes *et al* 2008, Schulte *et al* 2012, Saito *et al* 2017) and movement artefacts. Any external light, aside from the discrete wavelengths employed by the instrument, can interfere with measurement if it reaches the photodetector. Usually, ambient light pollution may cause offsets in the sensor or cause additional issues due to the flickering of lamps at 50 Hz (i.e. this frequency may vary across different countries), thus introducing unwanted noise in the PPG signals, which can possibly lead to erroneous SpO_2_ values. For this reason, pulse oximetry sensors should be optically insulated from any external light, and they should be positioned carefully to avoid ambient light reaching the photodetector. Using filtered photodetectors may alleviate the problem, but any light component in the red to the near-infra-red range of the spectrum (i.e. the effective range used in pulse oximetry) can still enter the sensor if not properly screened. Also, if pulse oximetry sensors are not properly positioned on the measurement tissue, mismatches in the red and infra-red absorption (penumbra effect) may cause false SpO_2_ readings. The penumbra effect re-emphasises the importance of correct probe positioning. Inappropriate probe placement leads to error readings, especially on the small fingers of neonates and infants. This effect occurs when the probe is not symmetrically placed, such that the pathlength between the two LEDs and the photodetector is unequal, causing one wavelength to be ‘overloaded’. Also, the emitted light can be projected tangentially to the detector (in reflectance probes), sometimes without crossing an arterial bed, leading to optical shunting. Repositioning of the probe often leads to improvement in saturation readings.

Furthermore, one of the ‘nemesis’ of pulse oximetry is sensor/tissue movement artefact and this should be limited during measurements. Good probe design and attachment as per application needs can help mitigate against this. Random extra-arterial movements can be easily recognised, but periodical movements, synchronous with the cardiac cycle, can be erroneously interpreted as ‘signal’ by the instrument and cause inaccurate SpO_2_ measurements. Advancements in signal processing techniques and technologies have helped to minimise the issue of movement artefacts in pulse oximetry (Chacon *et al* 2018, Louie *et al* 2018, Banik *et al* 2020). Some proprietary algorithms such as the Masimo Signal Extraction (SET^®^) (Goldman *et al* 2000) are already incorporated in modern pulse oximeters and are showing a robust estimation of SpO_2_ during motion (Hoskin and Granger 2001, Shah *et al* 2012).

Lastly, pulse oximeter readings can be impacted by intravenous and intradermal dyes (methylene blue, indocyanine green, patent blue, nitrobenzene and indigo carmine), which are used to identify anatomical structures (e.g. cancerous tissue, lymph nodes, etc) in diagnostic imaging or surgical operations. Usage of pulse oximeters during or shortly after the induction of these dyes has shown to have resulted in falsely low SpO_2_ values without actual desaturation. These errors occur as the pulse oximeter cannot distinguish between the absorption of light by intravenous dye and the absorption by haemoglobin species. For example, methylene blue has its peak spectral absorption at 668 nm, hence it absorbs most of the light emitted by the red emitter. This absorption is interpreted by the pulse oximeter as the presence of reduced haemoglobin, leading to inaccurate estimation of SpO_2_ (Sriganesh *et al* 2013, Ishiyama *et al* 2015). Therefore, caution must be taken when performing dye injections with concomitant pulse oximetry measurements.

In conventional practice, the effects of light scattering are accounted for via empirical calibration of the oximeter device, which appears to work well, but only up to a certain point (Webster 1997b). This is an unavoidable limitation of pulse oximeters as they can only be as accurate as their empirical calibration curves. These calibration curves are obtained from healthy subjects by measuring the red: infra-red ratio (5) while varying the inspired fraction of oxygen and, simultaneously, measuring SaO_2_ by blood-gas analysis and collecting dual-wavelength PPG signals. Understandably, researchers and pulse oximetry manufacturers are limited in the degree of hypoxaemia inducible in these volunteers, to an SaO_2_ of approximately 70%–75%. Therefore, the shape of the calibration curve below these levels must be extrapolated, with potential implications for the accuracy of pulse oximetry at low saturation levels. One of the limitations of this traditional calibration method is the limited range of oxygen saturation that can be acquired. Ethical issues prevent intentional desaturation of healthy subjects below a certain point due to risk of hypoxic brain damage. Also, another potential limitation of such calibration studies is perhaps the lack of balance between participating volunteers from all categories of skin pigmentation. Notably, the assumptions inherently made during an empirical calibration are valid only for a limited range of saturations and become invalid under extreme conditions.

## 3. Skin pigmentation as a limitation of pulse oximetry

Pulse oximeters use the optical properties of HbO_2_ and HHB in the visible and near infra-red regions to estimate blood oxygen saturation. Differences between arterial oxygen saturation measured by blood gas analysis and pulse oximetry was documented soon after their invention in the 1980s (Wang and Poh 1985, Cecil *et al* 1988, Gabrielczyk and Buist 1988, Ries *et al* 1989), yet the problem remains neither fully corrected nor fully understood (Sjoding *et al* 2021). A summary of the studies which compared the accuracy of pulse oximeters in groups of different race or skin pigmentation is provided in table 1. These were identified via a number of databases such as PubMed, Web of Science, and Google Scholar, using the following search terms/phrases: ‘pulse oximeter skin pigmentation’, ‘racial bias in pulse oximetry’, ‘pulse oximetry covid-19’, and ‘silent hypoxaemia’. Sources that were not identified using the search terms were retrieved from the references of identified publications. To be included, studies had to compare SpO_2_ against reference SaO_2_ measurements obtained via blood gas analysis on subjects with different skin pigmentation.

**Table 1.** Pertinent studies that compared the accuracy of pulse oximeters in groups of different race or skin pigmentation (available to access).

Study	No. subjects	Setting	Method for stratifying skin pigmentation	Statistical information	Findings
Wang *et al* (1985) (Wang and Poh 1985)	31 ‘pigmented patients’	Not available	Not available	Not available	Biox III oximeter gave readings that closely approximated oxygen saturation measurements from arterial blood, confirming the usefulness of non-invasive oximetry even in pigmented patients.
Gabrielczyk and Buist (1988)	21 (4 ‘racially pigmented’)	Post-cardiac surgery	Unclear	Bias ± SD (Overall): +0.6 ± 1.6%^a^	Statistically insignificant difference in accuracy of SpO_2_ between skin pigmentations.
Cecil *et al* (1988)	152 (136 White, 1 Asian, 15 Black)	Not stated	Scale of 1 to 3—Light, medium, or dark pigment	Bias (Overall): Nellcor, +0.59%. Ohmeda, −0.897%^a^	Both oximeters displayed a statistically significant, but clinically insignificant bias when compared with arterial blood oxyhaemoglobin. Greater accuracy in SpO_2_ measurements taken from Black patients was observed by the Ohmeda oximeter.
Ries *et al* (1989)	187 Distribution of racial groups not stated	Laboratory	Munsell colour system	Bias (Overall): Ohmeda, +1.4%. Hewlett Packard, −0.6%^a^	Readings were slightly less accurate in patient groups with darker skin, suggesting that dark skin colour may. affect the performance and accuracy of ear oximeters.
Jubran and Tobin (1990)	54 (29 Black, 25 White)	Intensive Care	Visual	Bias ± SD (White): 2.2 ± 1.8%^a^	SpO_2_ was less accurate and less precise in Black patients.
				Bias ± SD (Black): 3.3 ± 2.7%^a^	
Cahan *et al* (1990)	28 (22 White, 6 Black)	Hypoxia Laboratory	Unclear	Bias ± SD (White): 1.9 ± 5.1%^a^	Pulse oximetry values can be higher in Black subjects than in White subjects, especially at saturations below 80%.
				Bias ± SD (Black): 5.1 ± 4.6%^a^	
Lee *et al* (1993)	33 (22 Chinese, 6 Malaysian, 5 Indian)	Intensive Care	Race	Bias ± SD (Overall): +0.82 ± 2.6%^a^	SpO_2_ was most accurate in Chinese patients, and less accurate (greater overestimation) in Malaysian and Indian patients. It was also less accurate at low saturations and in patients with elevated bilirubin levels.
				Range: −4.9% − 10.5%	
Bothma *et al* (1996)	100 darkly pigmented adults	Intensive Care	Reflectance spectrophotometer	Bias (Overall): −1.0% − 1.2%^a^	The accuracy of pulse oximetry is not adversely affected by skin pigmentation, and it remains a useful oxygenation-monitoring device in darkly pigmented patients.
				LOA: −6.6% − 6.6% across all pulse oximeters	
				SD: 1.9%–2.4% across all pulse oximeters	
Adler *et al* (1998)	278 (34 Dark, 101 Intermediate, 143 Light)	Emergency Department	Munsell colour system	Bias ± SD (Light): +2.5 ± 4.6%^a^	The accuracy and precision of SpO_2_ was not affected by skin pigmentation. Signal quality was poorer in a great proportion of patients with dark skin pigmentation than intermediate or light.
				Bias ± SD (Intermediate): +2.8 ± 5.2%^a^	
				Bias ± SD (Dark): +2.2 ± 3.7%^a^	
Bickler *et al* (2005)	21 (11 Dark, 10 Light)	Hypoxia Laboratory	Race	Bias ± SD (Light): +0.37 ± 3.20%^a^	SpO_2_ was less accurate (greater overestimation) in Darkly pigmented subjects. This bias was greater at lower saturations, and differed between pulse oximeters.
				Bias ± SD (Dark): +3.56 ± 2.45%^a^	
Feiner *et al* (2007)	36 (17 Dark, 7 Intermediate, 12 Light)	Hypoxia Laboratory	Race	Bias range for intermediate and dark skin: +4.5%–+4.9% (SaO_2_ = 60% − 70%)^a^	SpO_2_ was less accurate (overestimation) in subjects with Dark and Intermediate pigmentation at lower saturations for five out of six combinations of pulse oximeters and probe types (the exception being the Masimo Radical with adhesive probe).
				+2.4%–+3.6% (SaO_2_ = 70% − 80%)	
Witting and Scharf (2008)	837 (577 African American, 260 White)	Emergency department	Self-report	Not available	African American group without hypoxaemia was associated with a 0.8-unit increase in SpO_2_ values, and without a change in precision. African American females and White females had an average SpO_2_ higher than African American men and White men, respectively. Clinicians should regard these findings with particular significance.
Foglia *et al* (2017) (Foglia *et al* 2017)	35 (14 Dark, 21 Light)	Infants with congenital heart disease	Munsell colour system	Bias ± SD (Overall): Nellcor, +3.9 ± 2.0% Masimo, +0.8 ± 2.4%^a^	No significant difference in SpO_2_ accuracy between patients with Dark and Light pigmentation.
Ebmeier *et al* (2018)	394 Distribution of racial groups not stated	Intensive Care	Fitzpatrick scale	Bias (Light versus Medium): −0.9%^a^	The accuracy of SpO_2_ was influenced by skin pigmentation and pulse oximeter model (Masimo underestimating, Philips overestimating).
				Bias (Light versus Dark): −2.4%^a^	
				LOA: −4.4%–4.4%	
Murphy and Omar (2018)	146 (6 Light, 111 Medium, 29 Dark)	Intensive Care	Massey New I mmigrant Survey skin colour scale	Bias ± SD (Overall): +1.64 ± 0.15 g dL^−1a^	The degree of skin pigmentation does not appear to influence the magnitude of bias, rather the increasing severity of illness and decreasing lower mean arterial blood pressure.
				LOA (Overall): −1.05 g dL^−1^–4.33 g dL^−1^	
Smith and Hofmeyr (2019)	220 12 Type I, 28 Type II, 69 Type III, 45 Type IV, 28 Type V, and 38 Type VI	Perioperative areas e.g. pre-assessment clinics, recovery rooms, operating theatres and intensive care units	Fitzpatrick scale	Bias (Overall): −0.55%^a^	Darker skin pigmentation showed no trend to an effect on the accuracy of oxygen saturation measured using portable fingertip pulse oximeter and a conventional bedside pulse oximeter.
				LOA: −3.25%–2.16%	
Sjoding *et al* (2021)	10,001 (1,326 Black, 8,675 White)	Intensive Care, and Inpatients receiving oxygen	Race	Not available	SpO_2_ was less accurate (overestimation) in Black than White patients. Hidden hypoxemia (arterial oxygen saturation of <88% and SpO_2_ of 92%–96%) was nearly three times as common in Black than White patients.
Valbuena *et al* (2021)	372 (65 Asian, 51 Black, 70 Hispanic, 186 White)	Intensive Care	Race	Incidence of occult hypoxaemia: 10.2% (White), 21.5% (Black), 8.6% (Hispanic), and 9.2% (Asian)	Hidden hypoxemia (blood gas arterial oxygen saturation of <88% and SpO_2_ of 92%–96%) was more common in Black patients than Asian, Hispanic or White patients.
Vesoulis *et al* (2021)	294 (124 Black, 170 White)	Neonatal Intensive Care	Race	Bias (White): +0.72%^a^	SpO_2_ was less accurate (overestimation) in Black than White patients. Hidden hypoxemia (arterial oxygen saturation of <85% and SpO_2_ ≥90%) was more common (although not significantly so) in Black than White patients.
				Bias (Black): +1.73%^a^	
Wiles *et al* (2021)	194 (34 Asian, 19 Black, 6 Other, 135 White)	Critical Care	Race	Bias (White): + 0.28%^b^	SpO_2_ was less accurate (overestimation) in Black, than Asian, than White patients (although no statistical test was used). Correlation between SpO_2_ and arterial oxygen saturation was lower in Black patients than Asian or White patients.
				LOA (White): −1.79%–2.35%	
				Bias (Asian): −0.33%^b^	
				LOA (Asian): −2.47%–1.80%	
				Bias (Black): −0.75%^b^	
				LOA (Black): −3.47–1.97	
Okunlola *et al* (2022)	491 (108 Dark, 383 Light)	Hypoxia Laboratory	Unclear	Not available	SpO_2_ was less accurate (overestimation) in Dark than Light to Medium skin pigmentations.
Wong *et al* (2021)	87,971 (1,919 Asian, 26,032 Black, 2,397 Hispanic, 57,632 White)	Intensive Care and other Hospital Wards	Race	Proportion of patients with hidden hypoxaemia:	The incidence of hidden hypoxemia (arterial oxygen saturation of <88% and SpO_2_ ≥88%) was greatest in Black, then Hispanic, Asian and finally White patients. It was associated with greater organ dysfunction 24 h later, and higher in-hospital mortality.
				White: 4.9%	
				Asian: 4.9%	
				Hispanic: 6.0%	
				Black: 6.9%	
Bangash *et al* (2022)	16,818 (81.2% White, 11.7% Asian, 4.0% Black, 3.2% Other ethnicities)	Hospital	Ethnicity	Relative to White patients:	Pulse oximetry tends to overestimate O_2_ saturation, and this is more pronounced in patients of Black ethnicity. These differences resulted in 6.1% versus 8.7% of White versus Black patients classified as normoxic on SpO_2_ who were hypoxic on the gold standard SaO_2_ reading.
				Bias (Asian): 0.5pp greater^a^	
				Bias (Black): 0.8pp greater^a^	
				Bias (Other): 0.3pp greater^a^	
Shi *et al* (2022)	6,505 (4,897 adults and 1,608 children)	27 out of 32 in hospital, none at home	15 studies measured skin pigmentation and 22 referred only to ethnicity	Bias ± SD (Light): −0.35 ± 1.49%^a^	Pulse oximetry may overestimate oxygen saturation in people with dark skin and people whose ethnicity is reported as Black/African American, compared with SaO_2_, although the overestimation may be quite small in hospital settings. The clinical importance of any overestimation will depend on the particular clinical circumstance.
	Distribution of racial groups not stated				
				LOA (Light): −1.87–4.09	
				Bias ± SD (Intermediate): −0.58 ± 1.47%^a^	
				LOA (Intermediate): −3.46–2.30	
				Bias ± SD (Dark): +1.11 ± 1.52%^a^	
				LOA (Dark): −3.27–2.58	
Baker and Wilson (2022)	75 (39 Black, 36 White)	Hypoxia Laboratory	Unclear	Bias ± SD (White): −0.05 ± 1.35^a^	There was no clinically significant difference in the accuracy or bias between Black and White subjects monitored with Masimo SET pulse oximetry.
				Arms (White): 1.35	
				Bias ± SD (Black): −0.2 ± 1.40^a^	
				Arms (Black): 1.42	
Crooks *et al* (2022)	2997 Distribution of ethnic groups not stated	Hospital	Ethnicity	Bias (White): +3.2%^a^	Pulse oximetry overestimated arterial oxygen saturations compared to blood gas measurement across all ethnicity groups when SpO_2_ measurements were below 90%, and underestimated these when SpO_2_ measurements were above 95%. However, individuals with Black, Asian or mixed ethnicity had a higher reading for oxygen saturation as measured by pulse oximetry than blood gas compared to individuals with a White ethnicity.
				Bias (Asian): +5.1%^a^	
				Bias (Black): +5.4%^a^	
				Bias (Mixed): +6.9%^a^	
Wiles *et al* (2022)	178 (126 White, 30 South Asian, 13 Black, and 9 other ethnic origin)	Intensive Care	Ethnicity	Bias (White): −0.25%^b^	Bias was greater in patients of non-White ethnic origin. The study also found that pulse oximetry is less accurate in patients diagnosed with COVID-19 and receiving mechanical ventilation.
				LOA (White): −4.75%–4.23%	
				Bias (South Asian): −0.96%^b^	
				LOA (South Asian):	
				−5.62%–3.71%	
				Bias (Black): −1.72%^b^	
				LOA (Black): −6.8%–3.36%	
				Bias (Other): −1.21%^b^	
				LOA (Other): −5.48%–3.05%	
Henry *et al* (2022)	26,603 (24,493 White, 1,263 Black, 574 Asian, 273 American Indian)	Intensive Care	Self-identification	Incidence of occult hypoxaemia: White (3.6%), Black (6.2%), Asian and American Indian (6.6%)	Occult hypoxemia is more common in Black patients compared with White patients and is associated with increased mortality, suggesting potentially important outcome implications for undetected hypoxemia. It is imperative to validate pulse oximetry with expanded racial inclusion.
Burnett *et al* (2022)	151,070 (16,011 Black, 21,223 Hispanic, 70,722 White, 8,121 Asian, 34,993 other)	Unclear	Self-identification	Bias ± SD (White): −0.20 ± 6.3%^a^	Self-reported Black and Hispanic race/ethnicity are associated with a greater prevalence of intraoperative occult hypoxemia in the SpO_2_ range of 92% to 100% when compared with self-reported White race/ethnicity.
				Bias ± SD (Hispanic): +0.5 ± 7.9%^a^	
				Bias ± SD (Asian): +0.2 ± 6.5%^a^	
				Bias ± SD (Other): +0.1 ± 5.9%^a^	
				Bias ± SD (Black): + 0.6 ± 9.1%^a^	
Fawzy *et al* (2022)	1216 (63 Asian, 478 Black, 215 Hispanic, and 460 White)	Referral centres and community hospitals	Self-identification	Bias (Relative to White Patients)^b^	Pulse oximetry overestimated arterial oxygen saturation among Asian, Black, and Hispanic patients (ethnic minority groups) compared with White patients with COVID-19. This contributes to unrecognised or delayed recognition of eligibility to receive COVID-19 therapies.
				Asian: −1.73%	
				Hispanic: −1.13%	
				Black: −1.23%	

LOA: Limits of agreement, SD: Standard deviation, pp: Percentage points

^a^ Bias= SpO_2 -_ SaO_2_

^b^ Bias= SpO_2 -_ SaO_2_.

### 3.1. Overestimation of arterial oxygen saturation

The mean difference between blood oxygen saturation measurements obtained by the two approaches is the ‘bias’, hence, ‘mean bias’ was referred to the average bias recorded within a racial subgroup. Out of the 28 studies identified in table 1, 22 studies found that SpO_2_ was overestimated in those with darker skin relative to reference SaO_2_ measurements obtained by blood gas analysis (Ries *et al* 1989, Cahan *et al* 1990, Jubran and Tobin 1990, Lee *et al* 1993, Adler *et al* 1998, Bickler *et al* 2005, Feiner *et al* 2007, Witting and Scharf 2008, Foglia *et al* 2017, Ebmeier *et al* 2018, Hunasikatti 2021, Sjoding *et al* 2021, Valbuena *et al* 2021, Vesoulis *et al* 2021, Wiles *et al* 2021, Wong *et al* 2021, Baker and Wilson 2022, Bangash *et al* 2022, Crooks *et al* 2022, Okunlola *et al* 2022, Shi *et al* 2022, Wiles *et al* 2022). Additionally, over half (57%) of the studies documented an increase in bias for subjects from all racial sub groups as they became less saturated (Cahan *et al* 1990, Jubran and Tobin 1990, Volgyesi and Spahr-Schopfer 1991, Lee *et al* 1993, Bothma *et al* 1996, Bickler *et al* 2005, Feiner *et al* 2007, Foglia *et al* 2017, Ebmeier *et al* 2018, Smith and Hofmeyr 2019, Vesoulis *et al* 2021, Wiles *et al* 2021, Wong *et al* 2021, Bangash *et al* 2022, Crooks *et al* 2022, Henry *et al* 2022, Shi *et al* 2022). For White subjects, bias values were observed between −0.35% and 3.2% and between 0.6% and 5.1% for Black subjects. Precision, which is given by the standard deviation (SD), was found to range between 1.8% and 6.3% for White subjects and between 2.7% and 9.1% for Black subjects. Evidently, bias and precision are seen to have a wider range in Black subjects relative to White subjects, which indicates that some pulse oximetry measurements are more accurate in individuals with fair skin.

In infants, a 1.5-fold overestimation of SpO_2_ was observed in Black infants relative to White infants for a SaO_2_ range between 85% and 100% (Vesoulis *et al* 2021). However, some biases in infants may be caused by cyanosis and not skin pigmentation (Mahle *et al* 2009, Ross *et al* 2014) although cyanosis is often observed differently in people with different skin tones (blue/purple in fair skin, grey/green in intermediate skin, and grey/white in dark skin). A mixture of fetal haemoglobin (HbF) and adult haemoglobin (HbA) could result in an underestimation of SpO_2_ of 3%–4%, or rotation of sensors every 12 h (to avoid skin injury) can cause inaccurate readings, as well as increase in melanin production following treatment in Black and Asian patients after phototherapy for jaundice patients (Vesoulis *et al* 2021). Such considerations are as important for rectifying bias caused in White subjects, specifically hypoxic patients. Hence, more work must be done on investigating bias at low arterial oxygen saturations (<90%), as this may provide some explanation about the overestimation of SpO_2_, and the occurrence of suboptimal function, which was observed two times more frequently in Black patients relative to White patients (Adler *et al* 1998).

Moreover, pulse oximeters were found to have a root-mean squared error (${A}_{\mathrm{rms}}$) of more than 3% in infants overall, with a greater discrepancy in Black infants (9.5% for Black infants and 8.9% for White infants) (Vesoulis *et al* 2021). Other studies recorded root-mean square values less than 3% (1.35% for White patients and 1.42% for Black patients using the Masimo SET) (Baker and Wilson 2022), (1.08% for White patients, 1.13% for Asian patients, and 1.56% for Black patients) (Wiles *et al* 2021). There is a greater urgency to address the huge inaccuracies observed in infants relative to adults, and to perform thorough testing of consumer marketed pulse oximeters, including new generation models (Rosychuk *et al* 2012), in order to prevent misleading interpretation of pulse oximeters readings, especially by home users (Okunlola *et al* 2022). The OpenOximetry.org Project is actively working to better understand the impact of skin pigmentation on oximeter accuracy and to develop new strategies to eliminate this source of error (Open Critical Care, n.d.). This includes laboratory testing on human subjects to determine the performance of existing oximeters used in a laboratory setting, and whether they perform differently in some patients in a clinical setting.

### 3.2. Incidence of hypoxaemia

As expected, the overestimation of SpO_2_ in Black subjects leads to an increased incidence of occult hypoxaemia (SaO_2_ < 88% yet SpO_2_ = 92%–96%) (Jubran and Tobin 1990, Michael *et al* 2020, Todd 2021, Valbuena *et al* 2021, Vesoulis *et al* 2021, Wong *et al* 2021, Burnett *et al* 2022, Henry *et al* 2022, Wiles *et al* 2022) ((Baker *et al* 2021) is a notable exception). Occult hypoxaemia was found to occur three times more frequently in Black subjects than in White subjects (Michael *et al* 2020, Valbuena *et al* 2021), two times more frequently in Black subjects than in White subjects (Burnett *et al* 2022), and in 9.2% of Black infants and 7.7% of White infants (Vesoulis *et al* 2021). Infants spend much of their hospitalisation period with oxygen saturations above 85%, so any small change in bias is critical, and can have adverse effects on mortality post-surgery (Nafiu *et al* 2020). Relying on pulse oximeters to triage patients and adjust supplemental O_2_ may place Black patients with increased hypoxaemia at risk (Todd 2021).

To correct for these levels of hypoxaemia amongst different racial groups, SpO_2_ targets of 92% and 95% were found to be reliable in predicting a satisfactory level of oxygenation in White and Black patients respectively (Jubran and Tobin 1990). These thresholds were determined for patients who recorded a PaO_2_ of 60 mmHg or above, however, it was more difficult to correct hypoxaemia while avoiding O_2_ toxicity for Black patients who recorded an average PaO_2_ level of 83 ± 31 mmHg (Jubran and Tobin 1990, Ralston *et al* 1991). In another study, these results were also consistent after the exclusion of patients with high COHb (seen to overestimate SpO_2_ (Adler *et al* 1998)), diabetes, and adjusting for age and sex and cardiovascular score, which indicates that the inaccuracies appear to be related to skin pigmentation. It is important to note that not all Black patients had occult hypoxaemia, but it is clear that there is a variation in risk due to race (Michael *et al* 2020), extending to greater organ dysfunction and higher in hospital mortality, even when age, sex, and sequential organ failure assessment score were adjusted (Wong *et al* 2021). Similarly in a recent retrospective study, Black patients diagnosed with Covid-19 were always at risk of unrecognised oxygen treatment eligibility based on pulse oximetry measurement (Fawzy *et al* 2022).

### 3.3. Methodologies

Conventionally, skin pigmentation is described by self-report or ethnic classification, without consideration of environmental factors affecting the quantity and distribution of melanin in the epidermis, such as exposure to ultraviolet B radiation. We identified some studies whose methods and/or outcomes did not comply with the United States Food and Drug Administration (FDA). According to regulations, 15% of the participant pool should be darkly pigmented. This requirement was met in (Wong *et al* 2021, Baker and Wilson 2022) (White pool = 65.5%, Black pool 29.6%, White pool = 55.44%, Black pool = 44.56%), but not in other studies (Guber *et al* 2019, Michael *et al* 2020, Valbuena *et al* 2021, Wiles *et al* 2021, Bangash *et al* 2022, Wiles *et al* 2022). Despite this, the inaccuracies seen in Black or darkly skinned subjects mentioned above did not change, although it was difficult to make conclusive statements in studies with a small number of volunteers (Gabrielczyk and Buist 1988).

The magnitude of the biases may have occurred due to a number of reasons. Firstly, some studies did not obtain simultaneous SpO_2_–SaO_2_ pairs as conducted in (Ebmeier *et al* 2018, Smith and Hofmeyr 2019, Baker *et al* 2021), since SaO_2_ measurements are prone to fluctuations over short periods of time. Instead, some measurements were taken a minimum of 4 min apart (Wiles *et al* 2021), 7.5 min apart (Bangash *et al* 2022), 10 min apart (Michael *et al* 2020), and up to 30 min apart (Crooks *et al* 2022). It is also important to consider that breathing variations, body temperature, anaemia, excessive motion, high levels of COHb and MetHb (Adler *et al* 1998), incorrectly applied probes/probe type (Feiner *et al* 2007), blood gas contamination by air in syringe (Vesoulis *et al* 2021), and perfusion status (Okunlola *et al* 2022), may have resulted in a small bias difference, especially in critically-ill patients (Wiles *et al* 2021).

Furthermore, only two studies (Ebmeier *et al* 2018, Guber *et al* 2019) used the Fitzpatrick scale (as recommended by the FDA) to categorise skin tones. Others chose to use the Munsell chart (Adler *et al* 1998, Feiner *et al* 2007, Foglia *et al* 2017) or mostly report race by self-identification (Witting and Scharf 2008, Vesoulis *et al* 2021, Bangash *et al* 2022, Wiles *et al* 2022). Only one study quantified pigment using reflectance spectrophotometry (Bothma *et al* 1996), which is the most objective method for classifying different skin colours.

### 3.4. Statistical significance

The majority of the data and information presented in this section share similar trends, however, not all these studies showed statistically significant differences. From table 1, 18 out of 30 studies (60%) concluded that their results showed a statistically significant relationship between skin pigmentation and overestimated SpO_2_ in subjects with darker skin (Cecil *et al* 1988, Gabrielczyk and Buist 1988, Ries *et al* 1989, Jubran and Tobin 1990, Lee *et al* 1993, Adler *et al* 1998, Bickler *et al* 2005, Feiner *et al* 2007, Sjoding *et al* 2021, Valbuena *et al* 2021, Vesoulis *et al* 2021, Wong *et al* 2021, Bangash *et al* 2022, Burnett *et al* 2022, Crooks *et al* 2022, Fawzy *et al* 2022, Okunlola *et al* 2022, Shi *et al* 2022, ). Some studies were either not able to conclude statistical significance due to limiting factors (e.g. sample size), or simply did not mention the statistical significance of the data (Wang and Poh 1985, Cahan *et al* 1990, Witting and Scharf 2008, Smith and Hofmeyr 2019, Wiles *et al* 2022). Additionally, there appears to be no clear guidelines for determining the level of clinically significant inaccuracy (or difference in accuracies). For instance, the differences in accuracy between groups were deemed to be clinically significant in Wiles *et al* (2021) and Volgyesi and Spahr-Schopfer (1991), and clinically insignificant in Bothma *et al* (1996), Adler *et al* (1998), and Baker and Wilson (2022), despite both sharing overlapping limits of agreements. Whilst it is beyond the scope of this narrative review to synthesise the results of different studies, we believe that the evidence indicates that at least some pulse oximeters are less accurate in people with darker skin pigmentation. Several recent large-scale studies have found lower accuracy and/or greater levels of occult hypoxaemia in people with darker skin pigmentation (Wong *et al* 2021, Bangash *et al* 2022, Burnett *et al* 2022, Crooks *et al* 2022, Fawzy *et al* 2022, Henry *et al* 2022, Shi *et al* 2022). We note that accuracy may differ between different pulse oximeter models, and future meta-analyses could consider this.

## 4. Natural and environmental factors of skin in relation to photoplethysmography (PPG)

### 4.1. Determinants of skin colour and appearance

Skin is a highly complex organ, and its colour and appearance are influenced by several factors. These include the presence of melanin, keratin, carotene, and haemoglobin, and other characteristics that differ with race such as hydration, texture, and homogeneity. The human skin is made up of multiple layers, each with their own distinctive optical properties that govern the absorption and scattering mechanisms of light.

Melanin is the primary determinant of skin colour in people with darker skin (Naik and Farrukh 2022). Carotene, keratin, oxygenated haemoglobin and water are other chromophores which lead to the differing absorption and reflection of light and hence varying shades of skin. The pink hue found in skin types I–II (from the Fitzpatrick scale) is produced by the combination of haemoglobin and oxyhaemoglobin, and the yellow-orange hue in skin types V–VI results from the combination of melanin and carotenes (Rawlings 2006). Natural melanin levels are determined by genetics, but can be influenced by quality of life, hormones, extrinsic and intrinsic aging, and skin pigment disorders, all of which alter the absorption coefficient and scattering coefficient of skin over time.

Melanin is produced in melanocyte cells, which are typically localised in the basal layer of the epidermis. The number of melanocytes does not account for the differences in skin colour, but rather, it is the variation in size, quantity, location, and distribution of melanosomes within melanocytes (where melanin pigment is synthesised) that contributes to the formation of skin colour (Szabó *et al* 1969, Toda *et al* 1972, Johnson *et al* 1998). In fair skin, melanosomes are smaller, found in clusters, degrade more quickly relative to more pigmented skin (Jothishankar and Stein 2019), and are synthesised in very acidic conditions (Ancans *et al* 2001, Fuller *et al* 2001, Watabe *et al* 2004). Contrastingly to darkly pigmented skin, melanosomes are larger, more dispersed along the basal layer, and synthesise at a higher pH of 6.8 (Jothishankar and Stein 2019).

Depending on the patient, it is important to select an appropriate measurement site to obtain reliable bio-optical data (Mantri and Jokerst 2022). This may differ from one patient to another, especially for those with dark skin pigmentation, as they are more prone to post-inflammatory hypopigmentation or hyperpigmentation relative to individuals with fair skin (Fitzpatrick *et al* 2018). Additionally, the colour of the nail bed in a Black person is much lighter than their skin, and so the behaviour of light through a finger (transmittance mode pulse oximetry) can differ relative to the behaviour of light through a palm (reflectance mode pulse oximetry).

### 4.2. The influence of skin pigmentation on the optical properties of skin

Dermatologists often use the Fitzpatrick scale, which describes skin colour based on a response to Sun exposure (Fitzpatrick *et al* 2018) which has been used to stratify skin pigmentation in experimental studies (Cahan *et al* 1990, Murphy and Omar 2018). The volume fraction of melanosomes in the epidermis layer of human skin ranges between 1% and 43% for very fair to very dark pigmentation, which in turn, represents a large variation in the average epidermal absorption coefficient that is also wavelength-dependent. Based on these skin types and their corresponding melanosome volume fractions as reported by Kanellis (2019) for fair, medium, and dark skin, the average absorption coefficient of the epidermis may be calculated using the following equation (Jacques 1998):

\begin{eqnarray*}\begin{array}{l}{\mu }_{{\mathrm{a}}\_\mathrm{epidermis}}\,\left[{\mathrm{cm}}^{-1}\right]\\ \,=\,\left({f}_{\mathrm{melanin}}\right)\left(6.6\times {10}^{11}\right)\left({\lambda }^{-3.33}\right)+\left(1-{f}_{\mathrm{melanin}}\right)\left(0.244+85.3{{\mathrm{e}}}^{\left(-\frac{\lambda -154}{66.2}\right)}\right),\end{array}\end{eqnarray*}

where *f*_melanin_ is the volume fraction of the epidermis occupied by melanosomes, and *λ* is the wavelength of light in nanometres (nm).

This yields to absorption coefficients of 0.72 mm^−1^, 4.20 mm^−1^, and 8.24 mm^−1^ for fair (*f*_melanin_ = 2.55%), medium (*f*_melanin_ = 15.5%), and dark skin (*f*_melanin_ = 30.5%) respectively using red light, and suggests that darkly skinned subjects may absorb nearly two times more red light than medium skin and over eleven times more than fair skin. Contrastingly, the absorptions of infra-red light by different skin types are more similar in magnitude, with absorption coefficients of 0.24 mm^−1^, 1.31 mm^−1^, and 2.59 mm^−1^ for fair, medium, and dark skin respectively. It is important to note that these values would vary between subjects in the same skin pigmentation group, and that also, these values be may not be entirely representative as the equations may be oversimplified. However, from a quantitative standpoint, these values may be indicative of the magnitude of change in the optical properties of the epidermis as melanin concentration vary.

Furthermore, the amount of light reflected from within or on the tissue surface contributes to the magnitude of light intensity output. Diffuse reflection is a type of reflection that arises from subsurface scattering of light in rough surfaces such as human tissue. The amount of diffuse reflection that takes place is influenced by the optical and geometric properties of human skin i.e., refractive index and angle of incidence (So-Ling and Li 2001). Fair skin types possess a low absorption coefficient and hence, more light can escape via diffuse reflectance. However, dark skin types have a high absorption coefficient, reducing the amount of diffuse reflectance, and tend to have a low scattering coefficient due to reduced blood supply to the skin. As a result, surface reflection relies on the combined effects of absorption and scattering based on skin pigmentation, hydration, sebum production, collagen network, and skin homogeneity.

Overall, it is important to distinguish between the characteristics of skin and their optical properties in order to understand the underpinning cause of overestimation of oxygen saturation in darkly pigmented subjects. It is possible that the distribution of melanin, colour, or racial differences, together or individually, are resulting in the inaccuracies observed in pulse oximetry measurement. The effect of each of these contributors on the optical properties of skin must be studied.

## 5. Clinical implications

We have established the effects of skin pigmentation on the accuracy of pulse oximetry measurement, and so it is vital to understand the impact of such outcomes to improve patient and consumer care as well as to inform clinicians of cautionary measures.

Pulse oximeters are widely used to identify and monitor signs of disease, and to help clinicians make informed diagnostic decisions. With the remote assistance of healthcare professionals, pulse oximeters have undoubtedly reduced the unnecessary admission of patients suffering from acute Covid-19 by allowing users to track their oxygen saturation levels at home. However, with the plethora of evidence suggesting their greater level of inaccuracy in patients with darker skin tones, we question the efficacy of this device in the recent global pandemic and in other clinical scenarios. Other clinical scenarios include the assessment and/ or diagnosis of sleep apnoea, newborn screening, hypoxaemia, and the administration of supplemental oxygen therapy.

Firstly, if pulse oximetry used in polysomnography is inaccurate in Black or Asian patients, related studies may need to be revisited (Philip *et al* 2021). For instance, several studies have documented Black males younger than 39 years and between 50 and 59 years to have a higher apnea-hypopnea index compared to White men of the same ages (where pulse oximetry measurements are used to assess the apnea-hypopnea index). Being a Black male younger than 40 years of age increased the apnea-hypopnea index by 3.21 breathing pauses per hour of sleep compared to a White man in the same age range with the same body mass index.

Secondly, pulse oximetry is often used to screen newborn babies to identify low blood oxygen saturations associated with critical congenital heart defects (Brown *et al* 2020). A baby can experience change in skin pigmentation from birth up until 12 weeks of age due to jaundice as a natural physiological response, or from breast milk, breast feeding failure, haemolysis, or inadequate liver function. Therefore, it may be difficult to obtain accurate measurements of SpO_2_, or even monitor changes in SpO_2_ if jaundice levels, and consequently skin colour, are inconsistent. It may be that the build-up of bilirubin in the blood has a greater impact on bias than melanin in infants, since the effect on bias is smaller than in adults (Vesoulis *et al* 2021).

Thirdly, fair skin types may inflict marginal influence on the pulse oximeter, with more significant errors in dark pigmented skin. As a result, its impact on clinical decision making could be significant at threshold values for diagnosis of hypoxaemia, with overestimated SpO_2_ leading to clinically important hypoxaemia remaining undetected and untreated. In addition, underestimated SpO_2_ readings have the potential to be harmful too, resulting in unnecessary treatment with oxygen (and the risk of hyperoxaemia) and wider impacts such as delayed hospital discharge (Shi *et al* 2022). In the context of profound hypoxaemia, clinicians should not rely on oximetry alone or on isolated readings (Knight *et al* 2022).

In clinical situations where oxygen is given despite normal SpO_2_, including carbon monoxide poisoning and states of increased cellular demand such as shock, sepsis, or major trauma, the need for supplemental oxygen is based on clinical judgement and severity of underlying condition (Cajanding 2021). Oxygen treatment is not without its risks, as we have already seen the higher prevalence of O_2_ toxicity in Black patients. Complications including nasal irritation, dry nose and throat, hyperoxia-related vasoconstriction, bacterial contamination of delivery systems, and lung injury, are common and can be potentially harmful (Cajanding 2021).

## 6. Recommendations

To address these issues in the short-term, inaccurate pulse oximeter models that are used in clinical settings or are publicly available for at home use must be identified, especially if their accuracy varies with skin pigmentation. Interestingly, some pulse oximeter models are cheaper yet perform similarly to more expensive and internationally standardised models when measuring hypoxaemia in healthy subjects (Lipnick *et al* 2016). Potentially, another temporary solution is to assign different SpO_2_ thresholds for the identification of hypoxaemia according to the patient’s skin pigmentation (Wong *et al* 2021) when using pulse oximeters that comply with FDA standards (i.e. ${A}_{\mathrm{rms}}\,$< 3%), provided that skin pigmentation is objectively quantified. Lastly, it may not be ideal to use pulse oximeters to monitor changes in SpO_2_ for oxygen administration based on saturation thresholds (Michard *et al* 2021), as there is research to indicate otherwise (Perkins *et al* 2003). Meanwhile, more studies should be conducted in real life settings to investigate the utility of pulse oximetry under a variety of conditions and circumstances.

It appears that the presence of melanin in the human epidermis may influence SpO_2_ estimation independently of blood oxygen saturation (Okunlola *et al* 2022). Melanin has the highest ability to absorb light in the visible and infra-red regions (characterised by ${\mu }_{{\mathrm{a}}}$) relative to all other skin chromophores and layers. Hence, it is a key contributor to the intensity of transmitted light in both transmittance and reflectance pulse oximetry (4). The majority of studies presented in this review have investigated the accuracy of transmittance finger pulse oximetry in medical settings. However, it would also be useful to explore the accuracy of oxygen saturation in subjects using smartphone applications, which rely on measurement of reflected light (Knight *et al* 2022). There has been some evidence to indicate that transmittance-based pulse oximeters possess a higher accuracy than reflectance-based pulse oximeters (Bangash *et al* 2022). Therefore, their use in medical settings may not be appropriate until algorithms to adjust for the reduced signal to noise ratio and other characteristics of phone cameras have been addressed (Luks and Swenson 2020).

A computational analysis of the effect of skin pigmentation on the accuracy of pulse oximetry in both PPG modes would be useful in determining the behaviour of light in a certain ROI, and the comparison of different outcomes (Huong and Ngu 2014). Across the years, Monte Carlo modelling has been a widely used tool for representing the stochastic nature of biological tissue, as it is a mathematical technique which involves modelling the probability of different outcomes with random variables. Although previous MC models (Wiles *et al* 2022) have briefly studied melanin between concentrations of 5% and 20% in their tissue model, there seemed to be no effect on calibration at a SpO_2_ of 40% (fetal pulse oximetry). Such models can be adapted and extended to look at the full range of melanin concentrations and observe the bio-optical changes for a broader range of arterial oxygen saturation levels. It is important to note here that the current equation (equation (10)) used to quantify the absorption coefficients of the epidermis, melanin, etc, can be oversimplified, and not sufficiently replicated in in-silico models. An alternative approach for modelling melanin at a more structural and molecular level can be achieved using the time-dependent density functional theory (Meng and Kaxiras 2008). Absorbance spectra can be simulated for all the constituent eumelanin monomers, which would be useful in understanding the influences of changes in melanin, particularly when interpreting results from *in vitro* experiments. However, this is a very timely procedure and therefore impractical from an engineering perspective and limited in the ability to optimise the sensor geometry, if required. Therefore, utilising a combination of modelling techniques must be considered when simulating melanin for a more accurate representation of skin pigmentation.

Furthermore, it may be useful to re-consider the wavelengths at which conventional pulse oximeters operate. There have been discussions on the influence of skin pigmentation on other applications such as PPG-based HR monitoring. They concluded that green light resulted in increased errors in individuals with darker skin tones due to the increased absorption of light by melanin at shorter wavelengths (Fine *et al* 2021), and that there were difficulties in choosing a wavelength which was insensitive to movement artefacts, poor skin perfusion and darker skin tones (Lemay *et al* 2021). As described earlier in figure 4, the estimation of SpO_2_ involves calculating the ratio of the normalised amplitudes of two pulse waves measured using red and infra-red light. Each normalised amplitude is calculated by dividing the pulse wave amplitude by the amount of light transmitted through the tissue. The higher concentration of melanin in patients with darker skin might affect the amount of light transmitted at both wavelengths, particularly red. Due to high absorption by melanin, less red light has been seen to travel through the finger, requiring a higher AC gain for better visualisation of the signal (Cahan *et al* 1990).

Recently, Hay*et al* (2018) found that use of a novel two infra-red pulse oximeter does not require empirical calibration. They expect that the accuracy of SpO_2_ measurement is to be greater than that of the red and infra-red pulse oximeter because of the small inter-person variability of the optical path lengths difference between the two wavelengths, resulting from the lesser impact of melanin in the near infra-red region. Another possible engineering solution is the use of multi-wavelength pulse oximeters, which consider the optical properties of multiple constituents in the region of interest. There is currently a prototype pulse oximeter which uses four short wavelengths (orange, red, blue, and green), and has shown to measure carboxyhaemoglobin saturation (SpCO) and methaemoglobin saturation (SpMet) more accurately than the Masimo Radical 7 (Chong *et al* 2017). However, further work is needed to test the prototype on a larger number of volunteers, and to ensure that they are racially diverse.

Race adjustments could potentially be built into future pulse oximeters, however, a manual adjustment based on the patient’s skin type may be the most feasible approach in the immediate short term until a concrete solution is found (Philip *et al* 2021). There is a need to collate PPG data from healthy dark-skinned subjects for saturations below and above 70% (Jubran and Tobin 1990, Calvin 1992, Lee *et al* 1993, Todd 2021, Holder and Wong 2022, Tobin and Jubran 2022) to observe the changes between PPG AC and DC with skin pigmentation. The correlation between SpO_2_ and R, must also be better established, as it may be unreasonable to assume a single relationship that relates R to SpO_2_ for all skin pigmentations. Perhaps pulse oximeters could use different relationships for different skin pigmentations, requiring either automatic recognition of skin pigmentation, or selection by the operator (Bickler *et al* 2005), although the latter could potentially lead to a considerable source of human error. Furthermore, calibration algorithms and calibration data employed in commercial pulse oximeters should be made available in order to improve awareness within the public and medical community of the possible bias introduced by calibration algorithm and data (Ferrari *et al* 2022).

Lastly, a key observation from the literature is the need to objectively quantify skin pigmentation. This can be achieved using RGB colour imaging (which indicates the level of red, green, and blue) (Verdugo-Naranjo *et al* 2022) and reflectance colourimetry, including the Mexameter^®^ (MX 18), Colorimeter^®^ (CL 400), and SkinColorCatch^®^ (previously DermaCatch). The latter measure a combination of melanin and haemoglobin index, L*a*b (L* = lightness, a* = red/green coordinate, and b* = yellow/blue coordinate), Individual Typology Angle (ITA), or L*C*h (L* = lightness, C* = chroma, h = hue angle) (Ly *et al* 2020). Interpreting these quantitative results is done by correlating the Fitzpatrick, ITA, and melanin index (Baquié and Kasraee 2014, Wilkes *et al* 2015, Agache *et al* 2017, Visscher 2017). Although there are a variety of methods to objectively measure skin pigment, Dermacatch^®^ has shown a significantly higher specificity and reproducibility than Mexameter^®^ in the measurement of skin pigmentation and erythema (Agache *et al* 2017). Overall, the connotations associated with the ‘racial bias’ in pulse oximetry can be re-defined, by establishing that this diagnostic inaccuracy may be due to differences in skin colour, texture, hydration, etc, rather than solely the quantity and quality of melanin in skin.

## 7. Conclusions

We have analysed the literature on the effect of skin pigmentation on pulse oximeter accuracy, and which reports that SpO_2_ is frequently overestimated in Black adults and infants, and in subjects with darker skin. As a result, these patients are more likely to experience occult hypoxaemia than White subjects, which may lead to delayed medical attention. This phenomenon has been revisited over 30 years after the introduction of pulse oximeters, since the reliance on such devices has increased during the Covid-19 pandemic, particularly for identifying hypoxaemia in the home. Although there are many questions regarding the accuracy of pulse oximeters possibly due to influence of skin pigmentation, they are still utilised in a variety of clinical applications and settings. Therefore, there is an urgent need to address this issue. We propose potential areas to investigate in the near future, such as the immediate identification of inaccurate pulse oximeters, the investigation of multi-wavelength pulse oximeters in subjects with different skin tones, obtaining more data from darkly skinned subjects to implement in-built calibration options, to objectively quantify skin pigmentation, and the development of computational models to predict differing bio-optical outcomes. Future work must include the development of pulse oximeter design and technology to eliminate bias associated with skin pigmentation, as well as all other known limitations.

## Data Availability

All data that support the findings of this study are included within the article (and any supplementary information files).
